# Shared and Differential Retinal Responses against Optic Nerve Injury and Ocular Hypertension

**DOI:** 10.3389/fnins.2017.00235

**Published:** 2017-04-26

**Authors:** Manuel Vidal-Sanz, Caridad Galindo-Romero, Francisco J. Valiente-Soriano, Francisco M. Nadal-Nicolás, Arturo Ortin-Martinez, Giuseppe Rovere, Manuel Salinas-Navarro, Fernando Lucas-Ruiz, Maria C. Sanchez-Migallon, Paloma Sobrado-Calvo, Marcelino Aviles-Trigueros, María P. Villegas-Pérez, Marta Agudo-Barriuso

**Affiliations:** Departamento de Oftalmología, Facultad de Medicina, Universidad de Murcia and Instituto Murciano de Investigación Biosanitaria Virgen de la ArrixacaMurcia, Spain

**Keywords:** glaucoma, chronic ocular hypertension, acute ocular hypertension, axotomy, Brn3a retinal ganglion cells, melanopsin retinal ganglion cells, cone photoreceptors, retinal nerve fiber layer

## Abstract

Glaucoma, one of the leading causes of blindness worldwide, affects primarily retinal ganglion cells (RGCs) and their axons. The pathophysiology of glaucoma is not fully understood, but it is currently believed that damage to RGC axons at the optic nerve head plays a major role. Rodent models to study glaucoma include those that mimic either ocular hypertension or optic nerve injury. Here we review the anatomical loss of the general population of RGCs (that express Brn3a; Brn3a^+^RGCs) and of the intrinsically photosensitive RGCs (that express melanopsin; m^+^RGCs) after chronic (LP-OHT) or acute (A-OHT) ocular hypertension and after complete intraorbital optic nerve transection (ONT) or crush (ONC). Our studies show that all of these insults trigger RGC death. Compared to Brn3a^+^RGCs, m^+^RGCs are more resilient to ONT, ONC, and A-OHT but not to LP-OHT. There are differences in the course of RGC loss both between these RGC types and among injuries. An important difference between the damage caused by ocular hypertension or optic nerve injury appears in the outer retina. Both axotomy and LP-OHT induce selective loss of RGCs but LP-OHT also induces a protracted loss of cone photoreceptors. This review outlines our current understanding of the anatomical changes occurring in rodent models of glaucoma and discusses the advantages of each one and their translational value.

## Introduction

The mammalian retina is an extension of the central nervous system (CNS) specialized to capture environmental luminous information. Light is transduced into electrical signals by the photo pigments (opsins) expressed by rod and cone photoreceptors or by the intrinsically photosensitive retinal ganglion cells (ipRGCs). The rod and cone signals are further elaborated by intermediate neurons located in the outer and inner plexiform layers and reach their way out of the retina through the retinal ganglion cell (RGC) population. RGCs constitute approximately less than 3% of all retinal neurons but are the only ones whose axons exit the retina to convey luminous information to the retinorecipient target regions of the brain.

Luminous information serves three main purposes; allows a nocturnal perception of very dim lights, a colorful daylight perception of bright lights, and regulates a number of autonomous behaviors. The two first purposes are the basis of our conscious image-forming vision and this luminous information is carried out by the general population of RGCs, which express Brn3a. Light-triggered autonomous behaviors are carried out by the ipRGCs that express the photopigment melanopsin and are the basis for a number of non-image forming visual reflex behaviors (Hattar et al., 2002, 2003) such as the pupillary light reflex, the photoentrainment of circadian rhythms and photic suppression of melatonin (Lucas et al., 2014).

The retina has been a preferred extension of the adult mammalian CNS to study plasticity, regeneration and degeneration because of its accessibility for experimental manipulation and its well-known anatomy and physiology (Aguayo et al., 1987; Bray et al., 1987, 1991). The capacity of adult mammalian CNS neurons for axonal regeneration (Munz et al., 1985), re-innervation of their appropriate targets (Vidal-Sanz et al., 1991; Avilés-Trigueros et al., 2000), synapse formation (Vidal-Sanz et al., 1987, 1991; Keirstead et al., 1989) and recovery of simple visual behaviors (Sasaki et al., 1996; Whiteley et al., 1998; Vidal-Sanz et al., 2002) has been investigated in the adult rodent retina (Aguayo et al., 1987; Bray et al., 1987, 1991).

Rodent RGCs share their location in the innermost retinal layer of the retina with the displaced amacrine cells, a population that overlaps RGCs in soma size (Villegas-Pérez et al., 1988, 1993) and is even larger than the RGC population itself (Nadal-Nicolás et al., 2015a). This has obliged the utilization of specific labeling techniques to identify RGCs and distinguish them from the displaced amacrine cells (Vidal-Sanz et al., 2000), including the use of retrogradely transported neuronal tracers applied to their axons or targets (Thanos et al., 1987; Vidal-Sanz et al., 1987, 1988) and specific RGC markers. Morphological criteria such as soma and dendritic arborisation size and levels of stratification within the inner plexiform layer, as well as electrophysiological responses to light stimuli and target region of the brain, may render over 30 different types of RGCs in the healthy rodent retina (Sun et al., 2002a,b; Coombs et al., 2006; for review see: Sanes and Masland, 2015). Many of these attributes change after injury and thus cannot be used to identify damaged RGCs (for review see Tribble et al., 2014). Molecular markers for RGCs are scarce, most do not label the entire population of RGCs and are downregulated in response to retinal injury (Chidlow et al., 2005; Lönngren et al., 2006; Agudo et al., 2008), thus rendering their use unreliable to identify RGCs. At present, Brn3a and melanopsin are two well-known molecular markers that identify most of the adult rodent (rats and mice) RGCs that conduct respectively, information related to image-forming and nonimage-forming visual functions. We have recently described that in the rat Brn3a is expressed by approximately 96% of the RGCs while the remaining 4% which do not express Brn3a is composed of approximately one half of the ipsilaterally projecting RGCs (1.3%) and the melanopsin expressing RGCs (m^+^RGCs) (2.6%) (Nadal-Nicolás et al., 2012, 2014; Galindo-Romero et al., 2013a; Valiente-Soriano et al., 2014). The differential expression of these two proteins, Brn3a and melanopsin, by the two functional types of RGCs makes double immunodetection a great tool to study in the same retinas the fate of these two distinct RGC populations upon injury and/or neuroprotection (Vidal-Sanz et al., 2015a,b; Agudo-Barriuso et al., 2016).

A progressive loss of RGCs and associated visual field deficits are a classic hallmark of the glaucomatous optic neuropathies (GON), a group of diseases that are the second leading cause of blindness in developed countries (Resnikoff et al., 2004). More recently, a number of additional features have been associated with GON, namely; characteristic defects in the nerve fiber layer, the optic disc and the optic nerve head (Quigley, 2011; Chauhan et al., 2014; Weinreb et al., 2014), as well as defects in the main subcortical and cortical visual targets (Yücel et al., 2003; Nucci et al., 2013). Moreover, other non-visually related areas of the cortex may also become affected (Frezzotti et al., 2014). In addition to RGCs, a number of reports have suggested that other non-RGC neurons are also affected in experimental or human GON retinas. Several studies have shown molecular, functional and structural changes in outer retinal layers (outer nuclear and outer segments) in humans (Nork et al., 2000; Barboni et al., 2011; Werner et al., 2011), non-human primates (Nork et al., 2000; Liu et al., 2014) and rodent models of glaucoma or ocular hypertension (OHT) (Mittag et al., 2000; Calkins, 2012; Fuchs et al., 2012; Pérez de Lara et al., 2014). These changes range from a diminution in the expression of opsins by photoreceptors to the severe loss of rods and cones with time (Ortín-Martínez et al., 2015).

The pathophysiology of GON is not fully understood, but much attention has been focussed on some of the most important risk factors and possible mechanisms; these include ocular hypertension, ischemia and axonal compression of the RGC axons within the initial segment of the optic nerve. Among the experimental rodent models of glaucoma there are two popular models that employ as a primary insult axotomy of the optic nerve or ocular hypertension.

The responses of RGCs to optic nerve injury have been studied extensively (Vidal-Sanz et al., 2000, 2007; Lindqvist et al., 2004; Jehle et al., 2008; Parrilla-Reverter et al., 2009a,b). Indeed, complete intraorbital optic nerve crush (ONC) or transection (ONT) are clean and reproducible models with relatively little inter-animal variability (Vidal-Sanz et al., 1987; Peinado-Ramón et al., 1996; Sobrado-Calvo et al., 2007; Parrilla-Reverter et al., 2009a; Sánchez-Migallón et al., 2011, 2016; Nadal-Nicolás et al., 2015a; Rovere et al., 2016a). Ocular hypertension tries to mimic one of the main risk factors of glaucoma and involves an increase of the intraocular pressure, but is a more complex model than axotomy and not as clean or reproducible (Salinas-Navarro et al., 2009c, 2010; Cuenca et al., 2010; Chidlow et al., 2011; Soto et al., 2011; Ortín-Martínez et al., 2015; Valiente-Soriano et al., 2015a,b; Rovere et al., 2016a). Recent studies have shown that m^+^RGCs are particularly resistant to a number of acquired or induced retinal diseases (Cui et al., 2015; Vidal-Sanz et al., 2015b; Agudo-Barriuso et al., 2016; Rovere et al., 2016a); but their response to glaucoma or ocular hypertension-induced retinal degeneration has not yielded homogeneous results (Li et al., 2006; González-Fleitas et al., 2015; Valiente-Soriano et al., 2015a,b). However, a surmounting body of recent evidence points that m^+^RGCs are severely affected in human glaucomatous retinas (Obara et al., 2016) as well as in animal models of ocular hypertension (Valiente-Soriano et al., 2015a,b).

There are several ways to raise artificially the IOP in rodents (for review see, Morrison et al., 2011; Vidal-Sanz et al., 2012, 2015a). Here, we will focus on a chronic and an acute model of ocular hypertension; the laser photocoagulation of limbar and perilimbar tissues and the anterior chamber cannulation. The main difference between these two models resides on the net increase of the IOP and on its duration. The chronic model consists of laser photocauterizacion of the limbar and perilimbar tissues to induce ocular hypertension (LP-OHT) (Levkovitch-Verbin et al., 2002; WoldeMussie et al., 2002). In albino rats LP-OHT results in a significant increase of the IOP that is already evident by 12 h, peaks at 48 h and remains significantly elevated for the first week, declining slowly to reach normal values by 3 weeks (Schnebelen et al., 2009; Salinas-Navarro et al., 2010; Ortín-Martínez et al., 2015; Valiente-Soriano et al., 2015b). In albino and pigmented mice, IOP levels raise above control values during the first 5 days, returning to basal values by 7 days after LP (Salinas-Navarro et al., 2009c; Cuenca et al., 2010; Valiente-Soriano et al., 2015a). The acute model of ocular hypertension (A-OHT) consists of cannulation of the anterior chamber with a needle connected to a saline reservoir that is elevated to increase the IOP for a short period of time; for instance in our Lab we increased IOP to 76 ± 3 mm Hg for 75 min (Rovere et al., 2016a; Wang et al., 2017). The fellow (contralateral) eyes showed normal levels of IOP (9 ± 1 mm Hg) at all time intervals studied.

Here we review some recent studies in our Laboratory on rodent models of GON, namely intra-orbital optic nerve injury and acute or chronic induction of ocular hypertension. We have compared the effects of optic nerve injury or ocular hypertension on the innermost (RGCs and all cells in the RGC layer) and outer (L- and S-cones) retinal layers. We have used imaging and counting techniques developed in our Laboratory to identify, count, and map in the same entire retinal wholemounts: (i) the general population of RGCs (non-melanopsin expressing RGCs, identified with Brn3a, Brn3a^+^RGCs); (ii) the population of intrinsically photosensitive RGCs (melanopsin expressing RGCs; m^+^RGCs); (iii) the population of cells in the RGC layer (identified by DAPI nuclear counterstaining); (iv) the nerve fiber layer of the retina (identified with neurofibrillary antibodies), and; (v) the L- and S-cone photoreceptor populations (identified with L- and S-opsin antibodies).

## Results and discussion

As we will see below, RGC damage in terms of quantitative and topographical loss differs between axotomy and OHT, as well as between Brn3a^+^ and melanopsin^+^RGCs. Furthermore, within each injury and RGC subtype, there are subtle differences among species and strain. Finally, neither insult causes the loss of non-RGC neurons in the ganglion cell layer (Ortín-Martínez et al., 2015; Nadal-Nicolás et al., 2015a) but ocular hypertension results in a secondary damage that reaches the outer retina (Ortín-Martínez et al., 2015).

### Temporal course of RGC loss: axotomy vs. OHT

**Figure 1:**
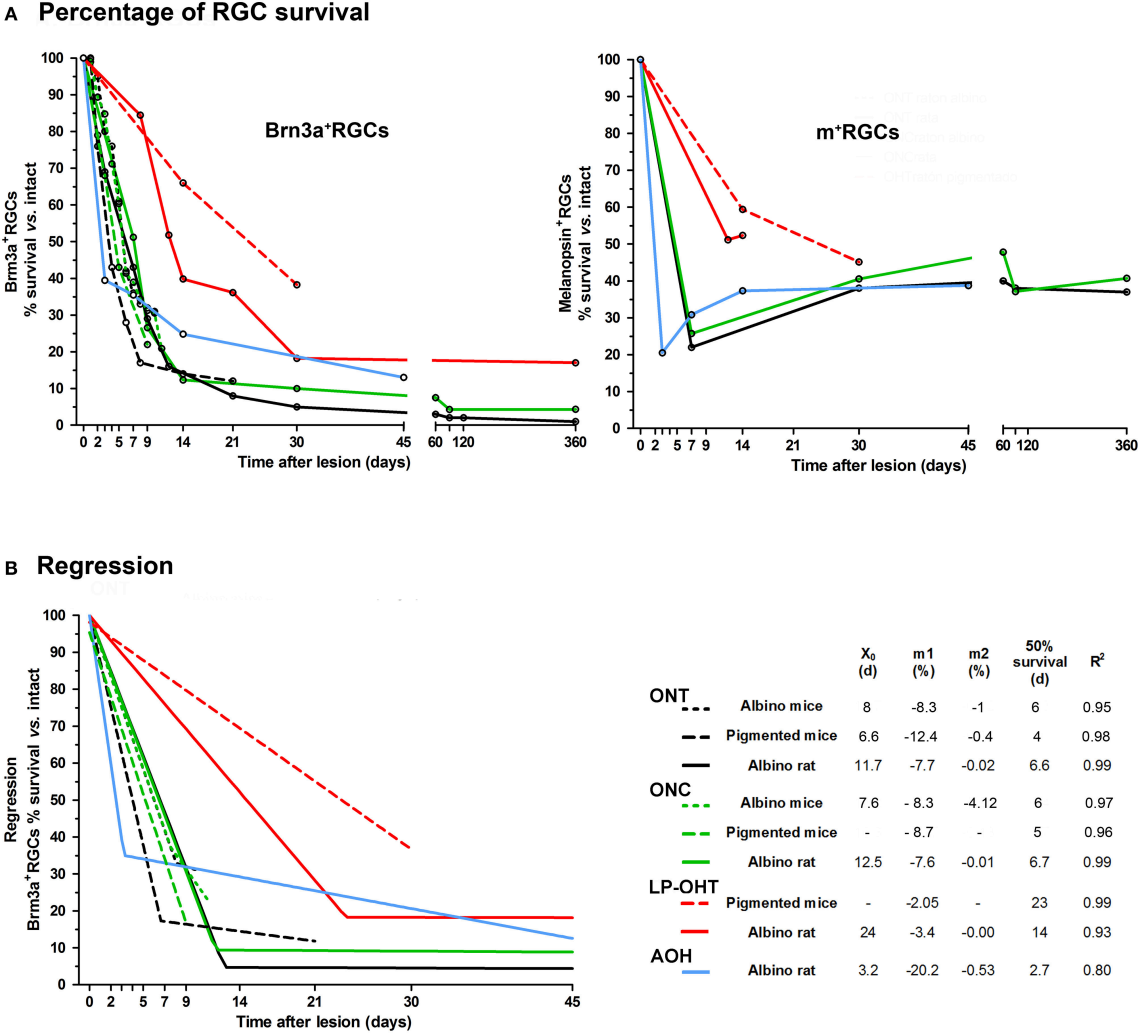
**Temporal course of RGC loss after axotomy or ocular hypertension. (A)** Graph showing the loss of Brn3a+RGCs (left) and melanopsin+RGCs (right) as percent of naive retinas vs. time post-lesion (days) after ONC, ONT, LP-OHT, or AOH in rats and mice. The open circles mark the time points of retinal analysis. Legend as in **(B). (B)** Graph showing the regression analysis of the loss of Brn3a+RGCs (percent of naive retinas, data in **(A)** vs. time post-lesion (days) after ONC, ONT, LP-OHT, or AOH in rats and mice. All courses adjust to either a segmental linear or to a linear regression. For ONT, ONC, and LP-OHT in rat the analysis was done with data points >45 days (see **A**) but the regression graph was cropped at 45 days for clarity. ONC, optic nerve crush at 0.5 mm from the optic head for mice, and 2 mm for rat. ONT: optic nerve transection at 0.5 mm from the optic nerve in both species. LP-OHT: ocular hypertension induced by laser-photocoagulation, in this model the increase of intra-ocular pressure lasts a week and rises on average up to 40 mmHg. AOH: acute ocular hypertension, induced by cannulating the anterior chamber, in this model the intra-ocular pressure was elevated to 76 mmHg for 75 min. X_0_ indicates the day when the first linear phase of RGC loss changes to a second, slower, one. m1 and m2 are the slopes of the first and second linear phases, respectively and show the daily loss of RGCs (in percent). Fifty percent survival is the time (days) when, according to the mathematical analysis, half of the RGCs are lost. *R*^2^ is the fitness of the regression. Albino mice: Swiss. Pigmented mice: C57/BL6. Albino rat: Sprague Dawley. These graphs were constructed using data from Salinas-Navarro et al. (2009c, 2010), Nadal-Nicolás et al. (2009, 2015a), Galindo-Romero et al. (2011, 2013b), Ortín-Martínez et al. (2015), Rovere et al. (2015, 2016a), Valiente-Soriano et al. (2015a,b), and Sánchez-Migallón et al. (2016).

To compare the time-course of RGC loss we gathered the results from our previously published work (Figure 1). We have estimated for each lesion, species and species strain the percentage of surviving Brn3a^+^− and melanopsin^+^RGCs at each time point, considering 100% the values in intact retinas (Figure 1A). All these values were obtained by quantifying the total number of RGCs identified in retinal whole-mounts. We performed an X,Y (time, survival) analysis using data in Figure 1A. We found that the loss of Brn3a^+^RGCs adjusts well to either a segmental linear or to a linear regression (Figure 1B), providing a mathematical model to compare more easily the damage caused by each lesion in each species and strain. These adjustments are useful as well to predict the percentage of survival (or loss) at a given time post-lesion, or *vice versa*, and thus, to design experiments within a possible window for therapeutic intervention.

#### Brn3a^+^RGCs: axotomy vs. OHT

As shown in the regression analysis, axotomy triggers the linear loss of Brn3a^+^RGCs in two sequential phases. During the first one, RGC death is abrupt and quick and lasts 7–12 days depending on the species, strain and the type of injury (Figure 1B, X_0_ values, for example, the response is quicker in pigmented than in albino mice). In the albino rat, during the first phase approximately 80–95% of RGCs die, and this is followed by a second slower phase that lasts up to 450 (Nadal-Nicolás et al., 2015a) or 600 (Villegas-Pérez et al., 1993) days, the latest time points analyzed. During the second phase, there is a protracted loss of RGCs (Villegas-Pérez et al., 1993), so that by the latest time points analyzed after the lesion approximately 1–3% of RGCs survive (Villegas-Pérez et al., 1993; Nadal-Nicolás et al., 2015a).

LP-OHT resulted in progressive loss of RGCs as well as in a number of typical findings observed in a mouse model of inherited glaucoma, the DBA/2J mice (Schlamp et al., 2006; Crish et al., 2010; Calkins, 2012; Pérez de Lara et al., 2014). These include: loss of RGCs in pie-shaped sectors with their base toward the retinal periphery and their apex toward the optic disc; early damage to the RGC axons near the ON head, and survival of RGCs with their axonal transport altered (both orthograde and retrograde) (Salinas-Navarro et al., 2009c, 2010; Cuenca et al., 2010; Chidlow et al., 2011; Soto et al., 2011; Valiente-Soriano et al., 2015a,b). The course of RGC loss after LP-OHT or axotomy differs in two main points: (i) in albino rats and mice the first phase of RGC death is longer (X_0_, 15–24 days) and less steep, and; (ii) RGC survival after LP-OHT is higher than after axotomy at the same time points. The main differences between rats and mice are that after the first phase of RGC death, LP-OHT does not cause a further loss in rats but it does in mice. However, we cannot discard that in mice RGC loss after LP-OHT stabilizes later than 30 days, the longest time analyzed in this species.

The loss of Brn3a^+^RGCs after A-OHT in rat also occurs in two phases. The first phase, in contrast with axotomy or LP-OHT, is very quick so that by day 3 approximately 65% Brn3a^+^RGCs are lost (Rovere et al., 2016b). Thereafter RGC loss progressed further to 70% by 14 days and to 87% by 45 days, but, as it occurs after LP-OHT, their survival percentage is higher than after axotomy (e.g., 13% at 45 days vs. the 5% found after ONT).

#### Melanopsin^+^ RGCs vs. Brn3a^+^RGCs: axotomy or A-OHT

When comparing the effects of axotomy or OHT on the survival of RGCs (Figure 1A) the first observation is a different survival ratio between Brn3a^+^RGCs and m^+^RGCs, both after axotomy or A-OHT. After axotomy, the number of Brn3a^+^RGCs in rats and mice diminishes linearly during the first 2 weeks, when less than 20–25% of the RGC population survive (Galindo-Romero et al., 2011, 2013b; Nadal-Nicolás et al., 2015a; Sánchez-Migallón et al., 2016; Rovere et al., 2016a). Thereafter their loss is slow and continuous, for example, 90 days after axotomy the survival of Brn3a^+^RGCs is approximately 5% (Nadal-Nicolás et al., 2015a). However, for rat m^+^RGCs the scenario is quite different: during the first days after injury their numbers diminish significantly, even more than those of Brn3a^+^RGCs, but later their survival is much higher (close to 40%) and moreover their numbers stabilize up to a year after axotomy (Nadal-Nicolás et al., 2015a). The lower number of rat m^+^RGCs identified early after the injury is caused by a transient down-regulation of melanopsin expression triggered by axotomy or retrograde Fluorogold tracing (Nadal-Nicolás et al., 2015b; Agudo-Barriuso et al., 2016) which impairs their immunoidentification. Later, melanopsin expression gradually recovers and it becomes clear that rat m^+^RGCs are more resilient than Brn3a^+^RGCs to axotomy or A-OHT (Nadal-Nicolás et al., 2015a; Rovere et al., 2016a). Retinal injury results in the modification of the expression of many RGC genes (Chidlow et al., 2005; Lönngren et al., 2006; Agudo et al., 2008, 2009; Agudo-Barriuso et al., 2013), including the transient downregulation of melanopsin (Nadal-Nicolás et al., 2015b), but RGCs have been shown to express both melanopsin and Brn3a long time after injury (Sánchez-Migallón et al., 2011, 2016; Galindo-Romero et al., 2013a; Nadal-Nicolás et al., 2015a). Thus, it is important to have in mind that in rats, melanopsin is not a good marker of viability in short-term experiments, and therefore long time experiments are required to assess accurately the survival of the population of ipRGCs.

Finally, in rats more Brn3a^+^RGCs survive after ONC than after ONT (Nadal-Nicolás et al., 2015a) probably because ONC is performed farther from the optic nerve head than ONT (Villegas-Pérez et al., 1993). However, the course of melanopsin down-regulation and m^+^RGC loss to these same lesions is comparable (Nadal-Nicolás et al., 2015a). This suggests that for m^+^RGCs, and in contrast to Brn3a^+^RGCs, neither the type of axotomy nor the distance from the optic nerve head at which the injury is inflicted have an influence in their response.

#### Melanopsin^+^RGCs vs. Brn3a^+^RGCs: LP-OHT

In rats and pigmented mice, Brn3a^+^RGCs and m^+^RGCs respond similarly to LP-OHT, and at the time points analyzed we have not observed neither a recovery of melanopsin expression nor a higher survival of m^+^RGCs than that of Brn3a^+^RGCs (Valiente-Soriano et al., 2015a,b). However, we have not carried out experiments at times longer than 15 days (rat) or 1 month (mice) and thus we do not know whether there is a recovery of melanopsin expression with longer survival intervals after LP-OHT.

### Topography of RGC loss

To visualize the distribution of RGCs in control and injured retinas we have used isodensity or neighbor maps generated from our quantitative data. Isodensity maps depict the density of a given cell population on the retina using a color scale (Salinas-Navarro et al., 2009a,b), and are useful to determine the topological distribution of highly abundant cells such as Brn3a^+^RGCs (Nadal-Nicolás et al., 2009, 2012) or cone photoreceptors (Ortín-Martínez et al., 2010, 2014). Neighbor maps illustrate the position of individual cells on the retina, the color assigned to each cell (dot) represents the number of cells around it (neighbors) in a given radius, and are useful to assess the topographic distribution of low abundance cells (i.e., m^+^RGCs, Galindo-Romero et al., 2013a; Nadal-Nicolás et al., 2014; Valiente-Soriano et al., 2014).

**Figure 2:**
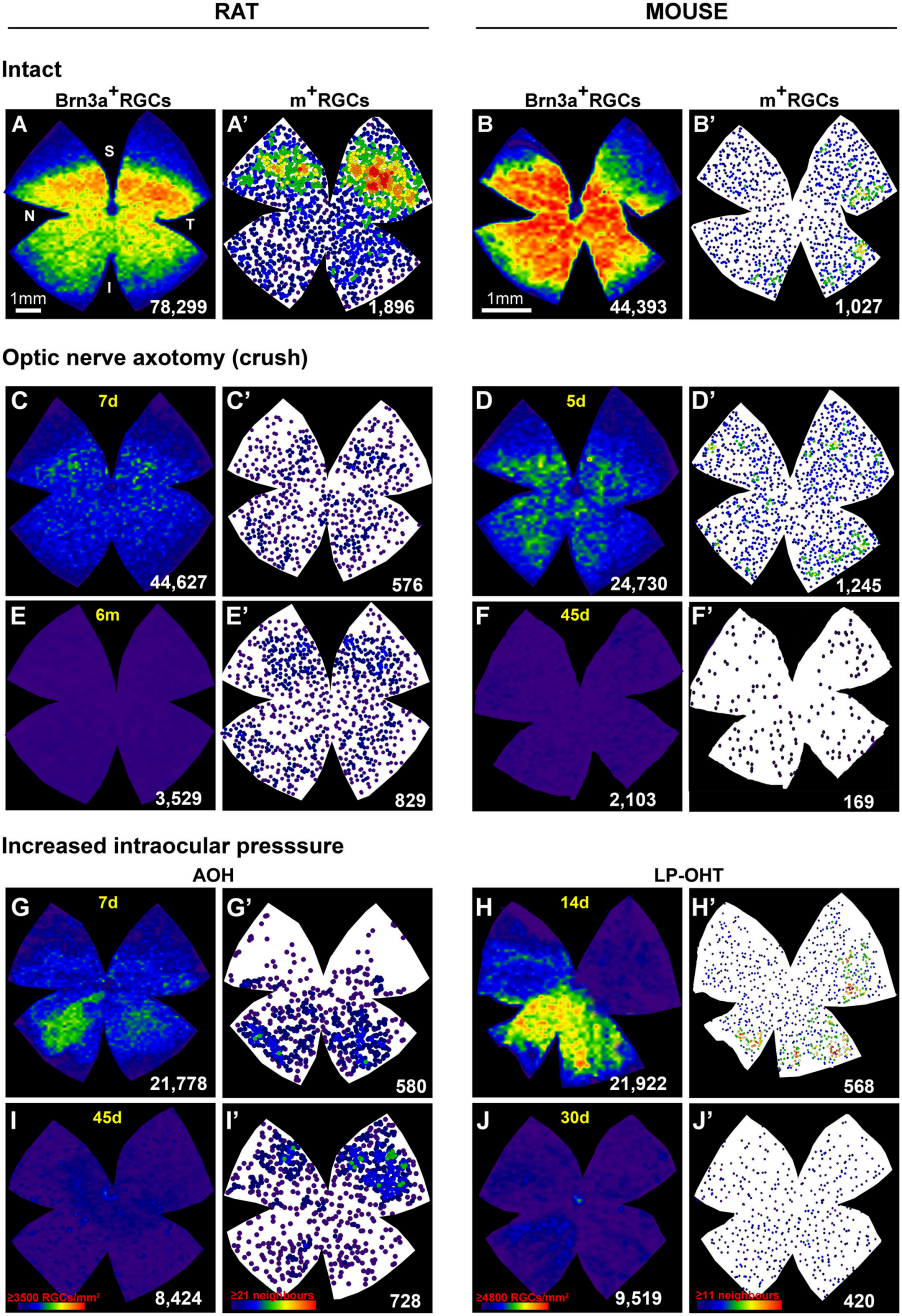
**Topography of RGC loss after axotomy or increased intraocular pressure**. **(A–J)** isodensity maps showing the distribution of Brn3a+RGCs intact **(A,B)** or injured **(C–J)** retinas from rat **(A–I)** or mouse **(B–J)**. (**A'–J')**: neighbor maps showing the distribution of melanopsin+RGCs in the same retinas as (**A–J). (C–F**'): axotomized retinas analyzed during the first phase of RGC death (**C,D**', 5 or 7 days) or long after the injury (**E,F'**, 45 days or 6 months). In both species axotomy causes a diffuse loss of both functional types of RGCs. Importantly, the survival percent of m+RGCs is higher than that of Brn3a+RGCs. In rat, shortly after the lesion **(C')** there are fewer m+RGCs than at 6 months **(E')** due to the transient downregulation of melanopsin (Nadal-Nicolás et al., 2015b). **(G–J')**: retinas analyzed after ocular hypertension (OHT) induced by either an acute increase of the intraocular pressure (**G,I**, 7 days and 45 days) or by laser photocogulation of the epiescleral and perilimbar veins **(H,J**, 14 or 30 days). OHT causes a sectorial or patchy loss of Brn3a+RGCs and a diffuse loss of m+RGCs. As observed for axotomy, m+RGCs are more resilient than Brn3a+RGCs. Again, in rat melanopsin expression recovers partially with time post-AOH (**G**' vs. **I**'). Isodensity maps depict the density of RGCs with a color scale that goes from 0 RGCs/mm^2^ (purple) to ≥3,500 (rat) or ≥4,800 (mouse) RGCs/mm^2^ (red). In the neighbors maps each dot represents a m+RGC and its color the number of neighbors around it from purple (0–1 in mouse or 0–2 in rats) neighbors to red (≥11 rat, or ≥21 mouse) neighbors in a radius of 0.22 mm (rat) or 0.165 mm (mouse). Below each map is shown the total number of RGCs quantified in their corresponding retina. Bar scale for rat in **(A)** and for mouse in **(B)**. N, nasal; T, temporal; S, superior; I, inferior; d, days; m, months; A–OHT, acute ocular hypertension; LP–OHT, laser photocoagulation induced ocular hypertension. These original isodensity maps were constructed using data from Salinas-Navarro et al. (2009c, 2010), Nadal-Nicolás et al. (2009, 2015a), Galindo-Romero et al. (2011, 2013b), Ortín-Martínez et al. (2015), Rovere et al. (2015, 2016a), Valiente-Soriano et al. (2015a,b), and Sánchez-Migallón et al. (2016).

The distribution of RGCs in intact (healthy) retinas is shown in Figures 2A,B'. In both species, the distribution of Brn3a^+^RGC and m^+^RGCs is complementary; Brn3a^+^RGCs are more abundant in the medial-central retina and m^+^RGCs in the periphery (Galindo-Romero et al., 2013a; Valiente-Soriano et al., 2014). Upon axotomy, the loss of Brn3a^+^RGCs is diffuse (Figures 2C–F) and while m^+^RGCs are lost across the whole retina, their population decreases predominantly in the dorsal retina (Figures 2C'–F'). Interestingly in rat the recovery of melanopsin expression that follows injury-induced downregulation of melanopsin occurs mainly in this area (compare panels C' and E').

Increasing the intraocular pressure, either by LP-OHT or A-OHT causes a sectorial or a patchy loss of Brn3a^+^RGCs (Figures 2G–J). This pattern of RGC loss differs between both models, after LP-OHT surviving Brn3a^+^RGCs are almost always found in pie-shaped sectors, with their widest part toward the retinal periphery and their vertex toward the optic disc, that are located predominantly in the ventral retina (Salinas-Navarro et al., 2009c, 2010; Valiente-Soriano et al., 2015a,b). A-OHT results in areas of low density of Brn3a^+^RGCs, with a much less reproducible geographical pattern (Rovere et al., 2016a), that is reminiscent of the pattern observed after transient ischemia of the retina induced by selective ligature of the ophthalmic vessels (Lafuente López-Herrera et al., 2002).

For their part, m^+^RGC loss after A-OHT or LP-OHT does not parallel the topography of Brn3a^+^RGC loss (Figures 2G'–J'). Indeed, m^+^RGCs loss is diffuse (Valiente-Soriano et al., 2015a,b; Rovere et al., 2016a) although more marked in the dorsal retina, as observed after axotomy. After A-OHT the recovery of melanopsin expression occurred, again, in the dorsal hemiretina (compare Figures 2G'–I').

**Figure 3:**
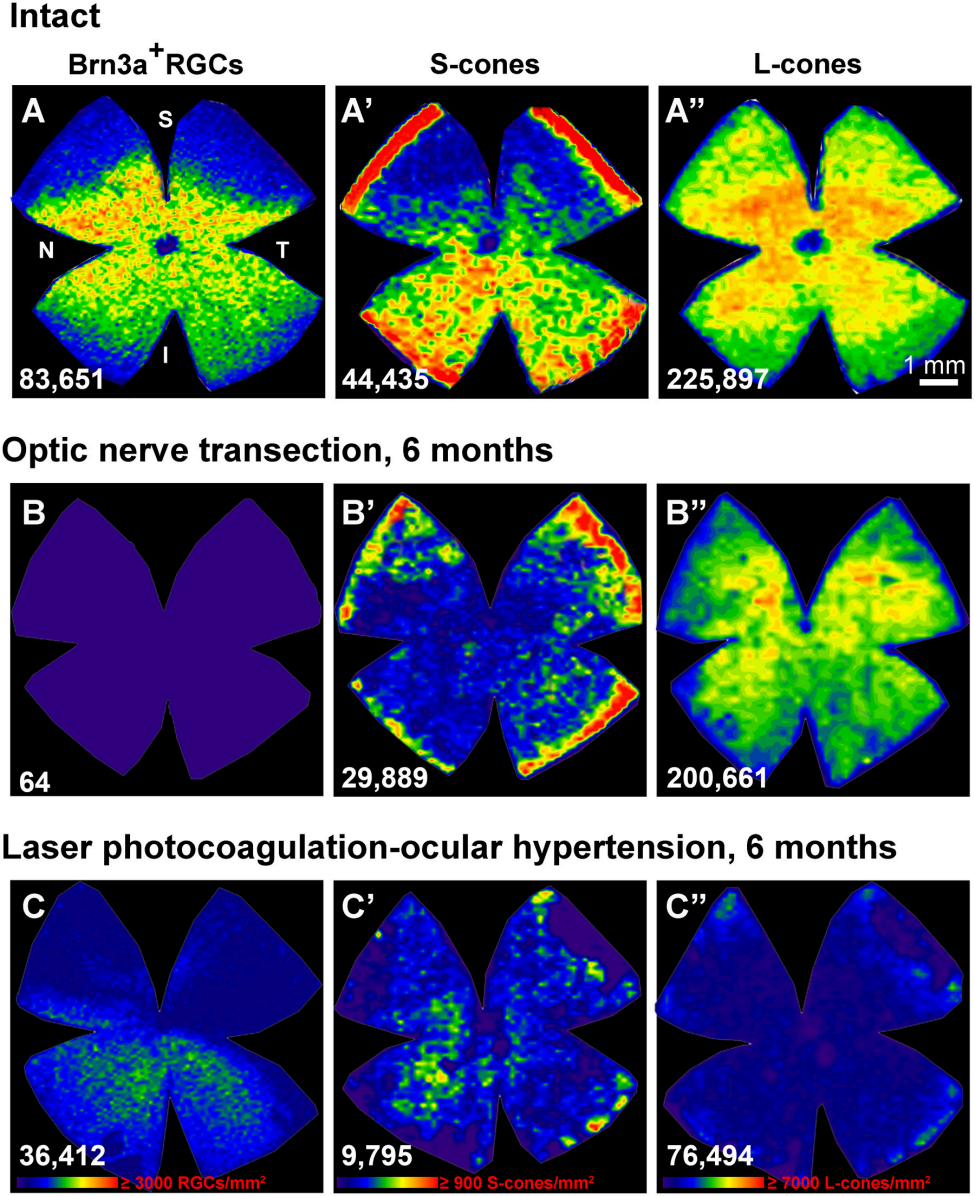
**Ocular hypertension but no optic nerve axotomy, causes the protracted loss of cone photoreceptors (A–C”)** : isodensity maps showing the topography of Brn3a+RGCs **(A–C)**, S-cones **(A'–C')** and L-cones **(A”–C”)** in the same retina within each group. **(A–A”)**: intact, **(B–B”)**: 6 months after axotomy, **(C–C”)**: 6 months ocular hypertension induced by laser photocoagulation. Isodensity color scale is found in **(C–C”)** and goes from 0 cells/mm2 (purple) to ≥3,000 RGCs/mm^2^, ≥900 S-cones/mm^2^ or 7,000 L-cones/mm^2^ (red). N, nasal; T, temporal; S, superior; I, inferior; d, days; These original isodensity maps were constructed using data from Ortín-Martínez et al. (2015).

It is tempting to speculate that the different topography of Brn3a^+^RGC loss reflects the nature of the injury itself (Vidal-Sanz et al., 2012, 2015a). Complete intraorbital ONC or ONT results in a diffuse loss throughout the retina with a marked absence of cells in areas or higher density, and this would be the result of lesioning the entire population of RGC axons within the ON head. Laser induced ocular hypertension results in a typical geographical pattern of pie-shaped sectors with their vertex toward the ON head (Salinas-Navarro et al., 2009c, 2010) that could be the result of damage to bundles of axons somewhere near the ON head, where they present their highest retinotopic arrangement (Guillery et al., 1995; Vidal-Sanz et al., 2012). Such a pattern of RGC loss is also observed for the very small proportion of Dogiel's RGCs that have their soma displaced to the inner nuclear layers (Nadal-Nicolás et al., 2015a). This particular pattern of retinal damage also appears reflected within the main retino-recipient areas of the brain. In rodents the vast majority of RGCs project to the contralateral superior colliculus (SC) (Salinas-Navarro et al., 2009a,b) and consequently ON axotomy results in complete SC denervation (Parrilla-Reverter et al., 2009a). LP-OHT results in a loss of synaptic terminals in concrete areas of the SC, reflecting their retinotopic distribution within the SC (Dekeyster et al., 2015; Valiente-Soriano et al., 2015a). AOH-induced RGC loss resulted in diffuse loss of Brn3a^+^RGCs with some areas showing fewer RGCs, but without a consistent pattern that could compare to that induced by axotomy or LP-OHT. As abovementioned, such pattern of RGC loss observed after A-OHT resembled the patchy RGC loss observed after transient ischemia of the retina induced by selective ligature of the ophthalmic vessels (see Figures 2, 3 in Lafuente López-Herrera et al., 2002), suggesting a possible predominant ischemic nature of the insult inflicted to these retinas.

### Retinal nerve fiber layer

The progressive degeneration of the intra-retinal RGC axons (the retinal nerve fiber layer, RNFL) following optic nerve injury has been studied with classic (Leoz et al., 1914; Ramón y Cajal, 1914) and modern (Vidal-Sanz et al., 1987; Villegas-Pérez et al., 1988) neurofibrillary staining techniques. Studies in rodents, using quantitative and qualitative methods to identify RGCs and their intra-retinal axons, indicated that the loss of intra-retinal axons was only evident long after the vast majority of RGCs had already died; following ONT or ONC (Parrilla-Reverter et al., 2009b) and LP-OHT (Salinas-Navarro et al., 2009c, 2010). These results highlighted the problem of estimating RGC survival based on the appearance of the RNFL (Vidal-Sanz et al., 2012). More recently, we have examined and compared the *in vivo* and *ex vivo* appearance of the RNFL with the actual population of surviving RGCs and their topological distribution at different survival intervals ranging from 3 to 120 days after ONT. These studies showed a delayed disappearance of the intra-retinal RGC axons in relation to the loss of their RGC somata (Rovere et al., 2015). Such a disagreement between the time course of RGC loss and RFNL thinning (Chauhan et al., 2012; Choe et al., 2014; Munguba et al., 2014) may be clinically relevant because measurements of the RNFL thickness are often used as an indirect index of RGC survival in glaucoma. However, extrapolation of these data from rodent models to human diseases requires certain caution since optic nerve injury in rodents results in a very rapid loss of RGCs, while human's glaucomatous degeneration is a chronic long-term disease with a progressive but slow time-course progression. Such a slow rate of RGC death is important because clearance of RGC bodies and axons is performed by microglial cells, and it is possible that after an acute and massive insult, such as optic nerve crush or transection, the clearance of axonal debris by microglial cells is slower than the clearance of RGC somas. Moreover, in rodents, ON injury induces RFNL swelling (Abbott et al., 2014; Rovere et al., 2015) and this could mask the actual thickness of the RNFL. This RNFL swelling could be explained by an alteration of the axoplasmic transport (McKerracher et al., 1990; Pease et al., 2000), or by an inflammatory response that includes macro and microglial proliferation in the RNFL (Salvador-Silva et al., 2000; Sobrado-Calvo et al., 2007; Ramirez et al., 2010; De Hoz et al., 2013; Galindo-Romero et al., 2013b; Rojas et al., 2014). Indeed, a recent study has shown that administration of non-steroidal anti-inflammatory drugs results in a decrease of the RNFL swelling that follows ONT (Rovere et al., 2016b).

### Responses of non-RGC neurons to optic nerve axotomy or OHT

Ocular hypertension and ON injury induce primary damage to RGCs, this is a well-established observation documented by the decrease of RGCs that survive such injuries. Whether other neurons in the ganglion cell layer (GCL) are also affected by these injuries has been unknown for some time, with the idea in mind of a possible protracted transneuronal degeneration following massive RGC loss. Recently, it has been shown that complete intraorbital ONT or ONC does not affect the survival of displaced amacrine cells (Nadal-Nicolás et al., 2015a). Detailed examination of the GCL with RGC markers and displaced amacrine cell markers in retinas whose optic nerves had been completely crushed or cut showed no significant diminutions in the total numbers of displaced amacrine cells for up to 15 months after the lesion, whereas surviving RGCs amounted to approximately 1% of the original population (Nadal-Nicolás et al., 2015a). Similarly, the GCL was examined short and long periods of time after the induction of OHT in adult rats (Ortín-Martínez et al., 2015) and mice (Valiente-Soriano et al., 2015a) to determine whether OHT resulted in specific loss of RGCs and not of other non-RGC neurons such as displaced amacrine cells. Retinas were immunolabelled with Brn3a to identify surviving RGCs and stained with DAPI to identify all nuclei present in the GCL. A topographic analysis of the GCL showed the typical pie-shaped sectors lacking Brn3a^+^RGCs, but with normal numbers of DAPI^+^ nuclei (see Figure 6 of Valiente-Soriano et al., 2015a; See Figures 3, 4 of Ortín-Martínez et al., 2015). These results documented that displaced amacrine cells did not die by OHT, as previously suggested (Jakobs et al., 2005; Kielczewski et al., 2005; Moon et al., 2005; Cone et al., 2010).

An additional long-standing controversy has been the issue of whether LP-OHT also induces damage to other neurons in the outer retinal layer (ORL) of the retina (Panda and Jonas, 1992; Kendell et al., 1995). Several studies in humans (Lei et al., 2008; Kanis et al., 2010; Choi et al., 2011; Werner et al., 2011), non-human primates (Nork, 2000; Nork et al., 2000; Liu et al., 2014) and rodent models of Glaucoma (Mittag et al., 2000; Heiduschka et al., 2010; Calkins, 2012; Fuchs et al., 2012; Fernández-Sánchez et al., 2014; Georgiou et al., 2014) suggested morphological and functional alterations of the ORL. Previous studies from this Laboratory (Salinas-Navarro et al., 2009c; Cuenca et al., 2010; Vidal-Sanz et al., 2012) had also indicated alterations in the ORL. More recently, we have examined whether there was cone-photoreceptor loss in adult rats short and long time intervals after LP-OHT induction and after ONT (Ortín-Martínez et al., 2015; Vidal-Sanz et al., 2015a) using retrogradely transported tracers and molecular markers to identify surviving RGCs and specific opsin antibodies to identify L- and S-cone-photoreceptors. In agreement with the quantitative data shown earlier, by 6 months after ONT almost all Brn3a^+^RGC have died, while the populations of L- and S-cone photoreceptors appears intact. This is in contrast to the situation observed after LP-OHT, the LP-OHT retinas showed approximately 70% RGC loss that was already apparent by 15 days but did not progress beyond 1 month and adopted the typical geographical pattern of pie-shaped sectors. In these retinas, the loss of L- and S-cones was first apparent by 1 month (35 and 20% loss, respectively) and progressed up to 6 months (80 and 65% loss, respectively). Moreover, there was a progressive downregulation of the rod-, L- and S-opsins to 60% of their normal values by 15 days and further decreases to less than 20% by 3 months after LP-OHT (see Figure 7 in Ortín-Martínez et al., 2015). Morphometrical analysis of paraffin-embedded cross-sections showed a significant reduction of the mean thickness of the outer nuclear layer (ONL) to approximately 2/3 of their normal values, and also a lack of cell nuclei in some areas of the ONL. Thus, indicating that in addition to L- and S-cone photoreceptors, rods had also degenerated (see Figure 8 in Ortín-Martínez et al., 2015). Overall, these data indicate that LP-OHT results in protracted degeneration of the outer retinal layers. The diffuse and patchy geographical pattern of L- and S-cones loss did not match the triangular pattern of RGC loss (Ortín-Martínez et al., 2015 and Figure 3). Because the retinal distribution of L- or S-cone loss does not parallel the sectorial loss of Brn3a^+^RGCs, it is tempting to suggest that the mechanism(s) that trigger the protracted death of cones is not related with the death of RGCs, and thus it is unlikely that such a loss of L- or S-cone photoreceptors reflects trans-neuronal retrograde cell death. It is possible that LP-OHT causes an additional insult, probably related to choroid ischemia, responsible for the protracted loss of L- and S-cones. In summary, our studies indicate that following LP-OHT there is selective loss of RGCs in the ganglion cell layer, but with time there is also a protracted degeneration of the outer retinal layers that result in both S- and L-cone-photoreceptor loss (Ortín-Martínez et al., 2015; Vidal-Sanz et al., 2015a). Thus, photoreceptor loss may constitute and additional feature associated with ocular hypertension and this may have important implications for human GONs.

## Concluding remarks

Following ONC or ONT there is rapid loss of RGCs followed by a more protracted loss of RGCs (Villegas-Pérez et al., 1993; Nadal-Nicolás et al., 2015a). The minute proportion of surviving Brn3a^+^RGCs contrasts with the much greater proportion of m^+^RGCs that survives by 6 months after ONC or ONT (approximately 41 or 37%, respectively) or by 15 months after ONT (approximately 39%). Therefore two main different biological responses of m^+^RGCs and Brn3a^+^RGCs against optic nerve injury emerge: (i) m^+^RGCs show a marked resilience against optic nerve axotomy reflected by the much smaller in magnitude amount of m^+^RGCs loss. (ii) There is no protracted progression of m^+^RGC loss beyond 1 month after injury as the population of m^+^RGCs remains stable from 1 to 15 months after injury (Nadal-Nicolás et al., 2015a). Following A-OHT there is also progressive loss of RGCs; by 2 or 6 weeks, 25 or 13%, respectively, of the Brn3a^+^RGC population survives in the retina. This is in contrast with the survival of m^+^RGCs for the same periods (37 or 39%, respectively), indicating as well a special resilience of these neurons to A-OHT. Following LP-OHT there is also progressive loss of RGCs but the proportions of surviving m^+^RGCs and Brn3a^+^RGCs (at 12 or 14 days for the rat, and at 14 or 28 days for the mouse) were comparable suggesting that rat and mouse m^+^RGCs do not show a special resilience against LP-OHT (Valiente-Soriano et al., 2015a,b). These results are in agreement with previous studies showing m^+^RGC loss and non-image forming visual functional deficits in humans (Pérez-Rico et al., 2010; Kankipati et al., 2011; Nissen et al., 2014; Obara et al., 2016) as well as in animal models of glaucoma (Drouyer et al., 2008; De Zavalía et al., 2011; Zhang et al., 2013).

ONT or ONC results in diffuse RGC loss throughout the retinas with greater losses in areas of higher densities. LP-OHT resulted in characteristic pie-shaped sectors lacking RGCs, with a greater loss in the ventral retina. A-OHT results in rapid loss of RGCs and its topological distribution was reminiscent of the patchy loss observed after transient selective ligature of the ophthalmic vessels for 90 min (Lafuente López-Herrera et al., 2002), suggesting that A-OHT results in a predominant ischemic insult to these retinas (Rovere et al., 2016a).

LP-OHT or ONT result within the GCL of the retina, at the time intervals studied of 6 months after LP-OHT and 15 months after ONT, in selective loss of RGCs but not of other non-RGC neurons, such as displaced amacrine cells. However, long after LP-OHT there is progressive degeneration of the outer retinal layers as observed functionally (Cuenca et al., 2010) and structurally (Cuenca et al., 2010; Ortín-Martínez et al., 2015). It is possible that LP-OHT could inflict outer retinal damage, independent of the RGC-axonal damage that would evolve slowly with time into a progressive degeneration of the rod, L- and S-cone photoreceptor populations. Because in our LP-OHT studies, important elevations of the IOP were present during the first 2 weeks, it is possible that choroidal insufficiency could be a distinct pathological event in this model (Nork et al., 2014; Ortín-Martínez et al., 2015).

Finally, to study the progression of glaucomatous optic neuropathy, animal models of OHT resemble better the pathophysiology of human glaucoma; they mimic not only the optic nerve damage but also the possible choroid damage and/or ischemic insult which are thought to be the underlying insults of the disease. Indeed, chronic OHT models show the characteristic sectorial loss of RGCs and ON damage as well as the protracted degeneration of the outer retina observed in human patients (Nork, 2000; Choi et al., 2011; Werner et al., 2011). Acute OHT models mimic an acute angle-closure glaucoma and may result in retinal ischemia leading to ON damage and RGC loss. These animal models of OHT show however, a high inter-animal variability and a poor correlation between the levels of IOP and the resulting damage, something that needs to be further investigated. On the other hand, optic nerve injury (axotomy) models are quite consistent in terms of topological, chronological and quantitative loss of RGCs. Thus, optic nerve crush or transection are quick and highly reproducible models for proof of concept assays to decipher injure-related molecular pathways and to test neuroprotective therapies.

## Author contributions

All authors have reviewed and approved the final version of this manuscript. Conceptualized and designed the experiments: MVS, CGR, FV, FN, AO, GR, MSN, FL, MCS, PS, MAT, MPV, and MAB. Performed the experiments: CGR, FV, FN, AO, GR, MSN, FL, MCS, and PS. Data acquisition and analysis: CGR, FVS, FN, AO, GR, MSN, FL, and MCS. Data interpretation and manuscript drafting: MVS, CGR, FV, FN, AO, GR, MSN, FL, MCS, PS, MAT, MPV, and MAB. Contributed reagents/materials/analysis tools: MVS, MAT, MPV, and MAB.

## Funding

Financial support for these studies was obtained from Fundación Séneca, Agencia de Ciencia y Tecnología Región de Murcia (19881/GERM/15), and the Spanish Ministry of Economy and Competitiveness, Instituto de Salud Carlos III, Fondo Europeo de Desarrollo Regional “Una Manera de Hacer Europa” (SAF2015-67643-P, PI16/00380; RD16/0008/0026, PI16/00031).

### Conflict of interest statement

The authors declare that the research was conducted in the absence of any commercial or financial relationships that could be construed as a potential conflict of interest.
